# Manual Development and Pilot Randomised Controlled Trial of Mindfulness-Based Cognitive Therapy Versus Usual Care for Parents with a History of Depression

**DOI:** 10.1007/s12671-016-0543-7

**Published:** 2016-05-25

**Authors:** Joanna Mann, Willem Kuyken, Heather O’Mahen, Obioha C. Ukoumunne, Alison Evans, Tamsin Ford

**Affiliations:** 1 Peninsula Medical School, University of Exeter, Oxford Institute of Clinical Psychology Training, University of Oxford, Oxford, UK; 2 Department of Psychiatry, Warneford Hospital, University of Oxford, Oxford, 0X3 7JX UK; 3 Mood Disorders Centre, University of Exeter, Exeter, UK; 4 NIHR CLAHRC South West Peninsula (PenCLAHRC), University of Exeter, Exeter, UK; 5 Peninsula Medical School, University of Exeter, Exeter, UK

**Keywords:** Mindfulness-based cognitive therapy, Depression, Parents, Children’s behaviour, Parenting

## Abstract

Parental depression can adversely affect parenting and children’s development. We adapted mindfulness-based cognitive therapy (MBCT) for parents (MBCT-P) with a history of depression and describe its development, feasibility, acceptability and preliminary estimates of efficacy. Manual development involved interviews with 12 parents who participated in MBCT groups or pilot MBCT-P groups. We subsequently randomised 38 parents of children aged between 2 and 6 years to MBCT-P plus usual care (*n* = 19) or usual care (*n* = 19). Parents were interviewed to assess the acceptability of MBCT-P. Preliminary estimates of efficacy in relation to parental depression and children’s behaviour were calculated at 4 and 9 months post-randomisation. Levels of parental stress, mindfulness and self-compassion were measured. Interviews confirmed the acceptability of MBCT-P; 78 % attended at least half the sessions. In the pilot randomised controlled trial (RCT), at 9 months, depressive symptoms in the MBCT-P arm were lower than in the usual care arm (adjusted mean difference = −7.0; 95 % confidence interval (CI) = −12.8 to −1.1; *p* = 0.02) and 11 participants (58 %) in the MBCT-P arm remained well compared to 6 (32 %) in the usual care arm (mean difference = 26 %; 95 % CI = −4 to 57 %; *p* = 0.02). Levels of mindfulness (*p* = 0.01) and self-compassion (*p* = 0.005) were higher in the MBCT-P arm, with no significant differences in parental stress (*p* = 0.2) or children’s behaviour (*p* = 0.2). Children’s behaviour problems were significantly lower in the MBCT-P arm at 4 months (*p* = 0.03). This study suggests MBCT-P is acceptable and feasible. A definitive trial is needed to test its efficacy and cost effectiveness.

## Introduction

Between 10 and 15 % of adults experience depression during their lifetime (Lépine and Briley 2011). Estimates of disability-adjusted life years due to unipolar depression are highest among those aged between 15 and 49 (WHO 2015), which are also typically the childbearing years.

Parental depression has been associated with increased intrusiveness and reduced sensitivity in parent-child interactions (Lovejoy et al. 2000) and with poorer outcomes for children, in terms of their emotional, behavioural, social (Davé et al. 2008; Goodman et al. 2011; Ramchandani et al. 2005; Velders et al. 2011) and cognitive development (Sharp et al. 1995). Interventions that prevent depression among parents may, therefore, improve parenting and break the inter-generational transmission of depression, with a potentially large impact on future depression-related burden of morbidity. There is evidence from the STAR*D trial that improving symptoms of parental depression can also result in improvements in children’s behaviour (Weissman et al. 2006).

Mindfulness-based cognitive therapy (MBCT) was designed to help people at risk for depressive relapse learn skills to stay well in the long term (Segal et al. 2002a). It is an 8-week group-based intervention that combines mindfulness practice and cognitive therapy so that people can recognise patterns of negative thinking and behaviour and learn more adaptive ways of responding. There is evidence that MBCT can reduce relapse rates for those who have experienced three or more episodes of depression (Piet and Hougaard 2011). In a qualitative study of parents who had participated in MBCT, many described being helped not only with their depression, but also with their parenting in terms of reduced emotional reactivity, enhanced empathy and greater involvement in parent-child interactions (Bailie et al. 2011). Previous qualitative studies (Allen et al. 2009; Bailie et al. 2011) have, however, emphasised that finding the time for the practices during the group intervention can be difficult and that specific modifications may be needed to improve the acceptability of MBCT for parents.

Mindfulness practices have also been added to existing parenting programmes with the aim of enhancing parent-child interactions (Coatsworth et al. 2010, 2014). There is evidence that a mindful parenting intervention for children with attentional and externalising problems improved these difficulties (Bögels et al. 2008, 2014; Oord et al. 2012). Bögels and Restifo (2013) described how mindful parenting activities can be brought into parenting and Bruin et al. (2015) concluded that a mindfulness training course for adolescents and their parents is feasible and described changes in adolescents’ quality of life and parental-reported competence in parenting. Considering the evidence for the impact of depression on parenting and children’s development and the potential of mindfulness training, there is a compelling rationale for a mindfulness-based intervention that aims to teach skills to help with the prevention of the relapse of parental depression *and* support mindful parenting.

We sought to integrate mindful parenting into the existing MBCT course, with a focus on emotional reactivity, enhanced empathy and parental involvement, whilst also ensuring that it was as acceptable and feasible for parents as possible. This was through the teaching of mindfulness skills which can be used in the context of parenting, but not the explicit teaching of parenting skills. The challenges parents face can elicit reactivity both in mood and in relationships with young children that may be key to the inter-generational transmission of depression (Hanington et al. 2010). There is also evidence that parenting programmes are more likely to be effective for young children (Evans 2006) and as such, we decided to adapt the existing intervention for parents of children aged between 2 and 12.

We aimed to adapt the existing MBCT group intervention for parents with a history of depression who also have young children (MBCT-P) so that the content also included mindful parenting activities (bringing mindfulness into the parenting context) and was more acceptable to parents. We aimed to explore the feasibility, acceptability and preliminary efficacy of the MBCT-P intervention, for parents, using the Medical Research Council’s (MRC) guidelines for developing complex interventions (MRC 2008). Efficacy was also explored in relation to children’s behaviour. We hypothesised that the course would be acceptable and feasible and that it would reduce symptoms of depression and parental stress, result in improvements in children’s behaviour compared with usual care and increase parental levels of mindfulness and self-compassion. The manual development stage involved interviews with two sets of parents: those who had taken part in standard MBCT groups and those who had taken part in MBCT groups in which the components to support mindful parenting were developed and piloted. The pilot randomised controlled trial (RCT) compared MBCT-P plus usual care versus usual care alone, to examine the accessibility and acceptability of the intervention and the feasibility of a definitive trial of MBCT-P.

## Method

### Participants

We recruited participants through family physicians, local health visiting teams, mental health services and advertisements placed in the community. Baseline and follow-up assessments took place either at participants’ homes or at the University of Exeter.

Randomisation was carried out using the web-based service Sealed Envelope (www.sealedenvelope.com), through a server with a secure data space, using encryption. Randomly permutated block sizes were used with stratification for anti-depressant use. Participants were randomised a month before an MBCT-P group. Nineteen parents were randomised to the MBCT-P arm. Two groups took place and parents were organised into the next group according to their time of randomisation. Four parents randomised to this arm did not attend any MBCT-P sessions, with one parent leaving the group once it had started. The first group, therefore, consisted of five parents and the second, nine parents.

**Fig. 1:**
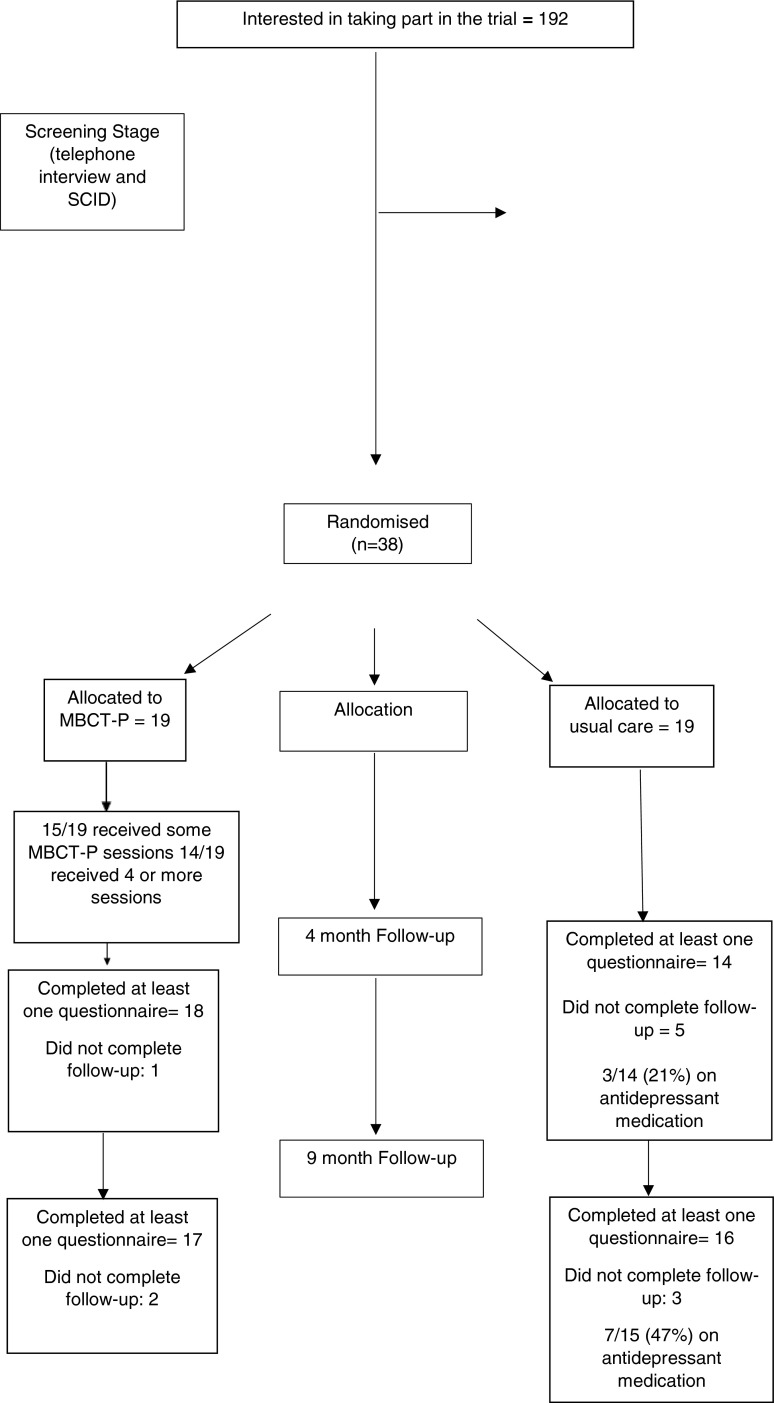
Consort flow diagram

The lead researcher (JM) conducted the randomisation. The lead researcher and seven other researchers (KL, PS, RV, CS, RH, KL and PH) conducted assessments (approximately 90 and 10 % respectively). Four researchers (PS, RV, KL, AH) coded assessments and all research staff other than the lead researcher were blind to trial arm status for those participants for whom they conducted or coded assessments. The structured clinical interviews were not conducted blind to randomisation status. Recruitment took place between March 2011 and April 2012, the 4-month follow-ups took place between July and October 2012 and the final 9-month follow-ups took place in early March 2013 (Fig. 1).

### Procedure

#### Development of MBCT-P Manual

The two MBCT-P manual development groups took place within an outpatient depression clinic. Seven parents took part in the first group and six in the second. The first author (JM) and therapists (AE and WK) met weekly following each group session to plan and review how to best adapt the MBCT programme to maintain the focus on the prevention of depression but also to incorporate mindful parenting. This included reviewing classes, which were videotaped, reviewing outcome measures and all available indicators of acceptability (direct feedback, engagement with practice and drop out).

In addition, three parents who had taken part in standard MBCT and nine parents who had taken part in the adapted MBCT-P course were asked about their experiences of what worked well and what changes might be required in a semi-structured interview with the researcher (JM) at the end of the 8-week course. All participants interviewed provided full informed consent. Inclusion criteria were to have experienced three or more episodes of depression, to be in full or partial remission from depression, and to have one or more children aged between 2 and 12 years. Exclusion criteria were current substance dependence, organic brain damage, current or past psychosis, current or past bipolar disorder, anti-social behaviour or persistent self-harm, already in receipt of psychological therapy, significant longstanding interpersonal difficulties that require specialist and longer-term psychological treatment and safeguarding concerns about children in the family.

#### Pilot Randomised Controlled Trial

A parallel group pilot randomised controlled trial assessed recruitment rates, level of attendance in the MBCT-P course and retention over follow-up. All parents who took part in the MBCT-P plus usual care arm of the trial were also asked for their views on the acceptability of the course and the trial procedures in a semi-structured interview. The trial is registered on the ISRCTN database: 98066741.

The trial compared MBCT-P plus usual care to usual care alone, over a 9-month follow-up period, in terms of depressive symptoms, depressive relapse, parental stress, levels of mindfulness, levels of self-compassion and children’s behaviour. ‘Usual care’ consisted of any care a parent received during the time of the trial. This care was accessed by the participants normal care providers and was independent of the trial. Participants in the intervention arm had access to this care as well. Inclusion criteria for the pilot RCT were the same as for the manual development, with the exception that the age range of children was narrowed to 2 to 6 years because parental depression may have a particular effect on younger children (NRC, Institute of Medicine 2009) and to narrow the range of parenting challenges faced by participants.

All participants provided full informed consent. The work was ethically approved (by the Bristol National Health Service committee, reference number: 10/H0106/81) and an independent Trial Steering Committee provided oversight of the trial.

#### Therapist Adherence and Competence

The two study therapists (AE and WK) co-ran the MBCT-P manual development groups and then individually ran the two MBCT-P pilot trial groups. Both of the therapists were mental health professionals and had previously received training from the developers of MBCT. They had also had their work extensively reviewed for both competency and adherence in previous trials (cf. Kuyken et al. 2008).

Adherence to the MBCT curriculum was measured using the MBCT-Adherence Scale (MBCT-AS) (Segal et al. 2002b). The MBCT-AS consists of 17 therapist behaviours which are rated on a 3-point scale (0 = no evidence of behaviour 1 = slight evidence of behaviour, 2 = definite evidence of behaviour). The items are summed to give a total score. Two subscale scores can also be calculated: one related to mindfulness behaviours and one to group CBT behaviours.

Therapist competence was measured using the mindfulness-based interventions teaching criteria MBI-TACS (Crane et al. 2012). There is evidence suggesting that this measure has good reliability and validity (Crane et al. 2013). The measure covers six domains: coverage, pacing and organisation of session curriculum, relational skills, embodiment of mindfulness, guiding mindfulness practices, conveying course themes through interactive inquiry and didactic teaching and the holding of the group learning environment. Raters provided a rating of each domain, using the following options: incompetent, beginner, advanced beginner, competent, proficient and advanced. An MBCT therapist independent to the trial and trained to a high level of adherence and competence provided the ratings based on two videotapes from each of the 8-week groups during the trial.

### Measures

The recruitment rate, rate of retention in MBCT-P group sessions and trial follow-ups were recorded. The following measures of depression, parental stress and children’s behaviour were completed by parents at baseline and 4- and 9-month follow-up. Total Beck Depression Inventory – II (BDI-II) score at 9-month follow-up was the primary outcome measure.

#### BDI-II

The BDI-II is a 21-item measure of depressive symptoms, with a range from 0 to 63 (Beck et al. 1996). In this trial, participants were asked to complete it in reference to a 1-week period because this measure was also completed weekly during the pilot trial to explore weekly change in symptoms. The BDI-II has been found to be sensitive to change, including in MBCT trials (Williams et al. 2008).

#### Parental Stress Index - Short Form (PSI-SF)

The PSI-SF consists of 36 items, summed to form three subscales and a total score that ranges from 36 to 180 (Abidin 1995). The PSI-SF has demonstrated sensitivity to change when used in previous trials of parenting interventions (cf. Hutchings et al. 2007).

#### Strengths and Difficulties Questionnaire (SDQ)

The SDQ is a 25-item measure of children’s behaviour and is composed of five subscales and provides a total difficulties score that ranges from 0 to 40 (Goodman 2001). The SDQ has demonstrated good internal consistency and retest reliability (Goodman 2001).

#### Five Facet Mindfulness Questionnaire (FFMQ)

The FFMQ is a 39-item questionnaire, measuring five facets of mindfulness (Baer et al. 2006). The scale as a whole has a total score with a range from 0 to 195. Baer et al. (2006) concluded that the FFMQ has several facets that contribute independently to the prediction of well-being and mediate the relationship between meditation experience and well-being. There is also evidence that the FFMQ is sensitive to differences in levels of mindfulness between participants who meditate regularly and participants who do not meditate (Baer et al. 2008).

#### Self-Compassion Scale (SCS)

The SCS is a 26-item measure consisting of seven subscales, the mean of each subscale was calculated and the sum of all of the subscales was totalled in this study, with a range of 1–30 (Neff 2003). Higher scores indicate higher levels of self-compassion. It has good internal consistency and test-retest reliability (Neff 2003).

#### Structured Clinical Interview

The Structured Clinical Interview for DSM IV (First et al. 2002) was used at baseline to establish whether parents had experienced three or more previous episodes of major depression, were in full or partial remission from depression and had no current substance dependence or bipolar disorder. It was then repeated at the 9-month follow-up to assess if participants had experienced any depressive episodes during the 9 months of the trial and if so, when these episodes occurred.

#### Semi-Structured Qualitative Interview

This interview took place with participants in the MBCT-P plus usual care arm, following the 9-month follow-up and took approximately 35 min. Participants were initially asked to describe their experience of the trial, how they found out about it, their expectations for the trial, and why they had decided to take part. This was followed by questions about the acceptability and accessibility of the group sessions and the measures and interviews used within the trial itself. Questions were designed to be as open as possible and were adapted where needed but all focused on the pre-specified topics described earlier.

### Data Analyses

#### MBCT-P Manual Development

Framework analysis was used to analyse the interviews with parents from standard MBCT groups and the MBCT-P pilot groups (Ritchie et al. 2003). The analysis was undertaken by JM whilst a second researcher coded two of the transcripts in order to provide a reliability check of the codes assigned (JH) and a third researcher (PH) used matrixes to form another categorisation that was compared to the initial categorisation (Ritchie et al. 2003). Discrepancies in the categorisation were discussed until consensus was agreed.

#### Pilot Trial

Characteristics and outcomes were summarised using means and standard deviations for quantitative variables and percentages for categorical variables. The outcomes were compared between the trial arms using the intention-to-treat principle with participants analysed according to the trial arm they were allocated to regardless of the treatment actually received. Complete case analysis was used where we included only those participants that provided outcome data at follow-up. No data were imputed. All pre-test scores were adjusted for in the analyses. Linear regression was used to compare quantitative outcomes between the trials arms, adjusting for baseline (pre-test) imbalances on the outcome measures. The Chi-squared test was used to compare the percentage that remained well at 9 months. Baseline BDI-II scores for parents who did and did not complete the 4-month follow-up, per trial arm, are reported.

## Results

### MBCT-P Manual Development

Participant demographics are shown in Table 1. In the manual development groups, attendance was high; one participant, respectively, attended 3, 4, 5 or 6 sessions whilst three attended 7 sessions and six attended all 8 available sessions. It was decided following a discussion with clinic staff, to include one parent who had two teenagers, the eldest being 14, in order to maximise the number of parents participating and ensure it was feasible for one of the MBCT-P groups to take place.

**Table 1:** Demographic characteristics of participants in the MBCT-P manual development phase

Characteristic	Qualitative interviews	MBCT-P groups
Number of participants^a^	12	13
Female, *n* (%)	10 (83)	13 (100)
White, *n* (%)	12 (100)	13 (100)
Age of parent in years, median (range)	46.5 (40 to 51)	43 (40 to 51)
Age of youngest child in years, median (range)	3.8 (0 to 14)	5 (0 to 14)
Number of children, median (range)	2 (1 to 3)	2 (1 to 3)

^a^Participants who took part in the qualitative study were from standard MBCT groups (three parents) and the MBCT-P groups (nine parents)

Parents’ feedback covered a range of topics including why they had decided to attend the group, their expectations for the group, the usefulness of the group and using mindfulness in parenting. Categories were produced using this feedback, for example, ‘view of the group’ and ‘difficulties with practice’. Categories were also produced which focused upon changes to be made to the course, for example, ‘course content, materials and environment’ and ‘course length and timings’. All feedback was considered in the context of the group in which parents had participated (MBCT or MBCT-P) to allow changes to be made to the manual as appropriate. For a more detailed description of the categorisation process, please see Mann (Mindfulness-based cognitive therapy for parents with recurrent depression, unpublished thesis), available from the corresponding author.

We kept the manual as close to the original MBCT manual (Segal et al. 2002a) as possible, but added adaptations that would assist parents’ participation in an MBCT group and introduce elements to promote mindful parenting (Kabat-Zinn and Kabat-Zinn 2008). Following discussion with the MBCT therapists and feedback from parents who had taken part in the MBCT groups, adaptations were made to the manual. The adaptations made were the option for parents to choose shorter meditation practices to make it more feasible for them to establish a regular mindfulness practice and the adaptation of existing exercises within the course so that they had a parenting context, for example, following session 2, parents recorded pleasant activities that involved their children. There was also the addition of mindful parenting activities in the classes and homework. These were graded throughout the 8-week course, so that parents began with small and manageable mindful parenting activities (e.g., mindfully watching their child whilst asleep) and worked towards acting mindfully during a difficult situation with their child (e.g., when both parent and child are tired and the child is demanding).

Parents who had taken part in the two MBCT-P pilot groups provided feedback which was used to assess the acceptability of the manual and whether any additional changes needed to be made to the course. Parents described the course as acceptable and only small changes were made following the MBCT-P pilot groups; for example, some additional examples of mindful parenting activities were added in. This manual was then used in the pilot trial, comparing MBCT-P plus usual care to usual care alone. The full finalised manual is available from the corresponding author.

## Pilot Randomised Controlled Trial

### Acceptability and Feasibility of MBCT-P

Out of a total of 192 parents who expressed an interest in taking part in the trial, 99 participants were screened and 38 were recruited and randomised (see Table 2). The most significant barrier to trial participation was difficulties with the timing of the sessions. The second MBCT-P group was, therefore, run in the evening, which proved easier for many parents. In the pilot trial, 14 of the 19 (74 %) participants randomised to MBCT-P, attended four or more session and 4 out of 19 participants randomised to MBCT-P did not attend any sessions (21 %). At 9-month follow-up, 17/19 (89 %) parents completed at least one outcome measure in the MBCT-P plus usual care arm and 16/19 (84 %) in the usual care arm. The number who completed each measure at each time point is recorded in Table 3.

**Table 2:** Baseline characteristics of the MBCT-P and usual care arms in pilot RCT

Characteristic	MBCT-P (*n* = 19)	Usual care (*n* = 19)
Female, *n* (%)	18 (95)	18 (95)
White, *n* (%)	19 (100)	18 (95)
Age of participant in years, mean (SD); range	37.1 (5.3); 27 to 48	35.3 (4.9); 27 to 43
Age of trial child in years, mean (SD); range	4.1 (1.3); 2 to 6	4.2 (1.4); 2 to 6
Number of children participant has, mean (SD); range	2.2 (1.2); 1 to 5	2.2 (0.8); 1 to 4
Religion^a^		
Christian, *n* (%)	10 (53)	6 (32)
Muslim, *n* (%)	0 (0)	1 (5)
Atheist, *n* (%)	0 (0)	1 (5)
Agnostic, *n* (%)	1 (5)	0 (0)
Not stated, *n* (%)	4 (21)	1 (5)
Marital status		
Single, *n* (%)	1 (5)	1 (5)
Cohabiting, *n* (%)	2 (11)	1 (5)
Married, *n* (%)	14 (74)	13 (68)
Divorced, *n* (%)	2 (11)	2 (11)
Separated, *n* (%)	0 (0)	2 (11)
Level of education		
Some school qualification, *n* (%)	0 (0)	3 (16)
College of vocational qualification, *n* (%)	6 (33)	6 (32)
Degree or professional qualification, *n* (%)	12 (67)	10 (53)
Standard occupational classification^b^		
Major groups 1 to 3	6 (32)	7 (36)
Major groups 4 to 7	5 (26)	8 (43)
Full-time parent	7 (37)	4 (21)
Student	1 (5)	0 (0)

^a^Four participants in the MBCT-P arm and 10 participants in the usual care arm marked their religion as n/a

^b^Rated according to the Standard Occupational Classification (SOC) 2000 (ONS 2000)

**Table 3:** Mean scores for each outcome at each time point, per arm of the trial

Measure	Baseline				4-month follow-up				9-month follow-up			
	MBCT-P		Usual care		MBCT-P		Usual care		MBCT-P		Usual care	
	Mean (SD)	*n*	Mean (SD)	*n*	Mean (SD)	*n*	Mean (SD)	*n*	Mean (SD)	*n*	Mean (SD)	*n*
Beck Depression Inventory	13.0 (10.0)	19	9.51 (8.1)	19	8.1 (9.1)	18	6.9 (9.0)	14	4.6 (4.8)	17	11.3 (10.8)	16
Parenting Stress Index - short form	84.2 (17.6)	19	77.8 (16.4)	14	73.2 (13.6)	13	84.1 (25.5)	9	69.6 (13.4)	15	74.1 (15.9)	16
Strengths and difficulties questionnaire	8.6 (3.9)	18	10.3 (5.0)	13	7.1 (3.0)	18	8.8 (4.0)	10	7.7 (3.6)	17	10.3 (5.9)	16
Five facet mindfulness questionnaire	112.9 (15.5)	19	119.79 (14.4)	14	127.6 (19.7)	18	124.3 (22.0)	11	136.2 (17.6)	17	122.7 (25.1)	16
Self-compassion questionnaire	13.96 (13.10)	19	14.51 (3.20)	14	17.35 (3.56)	18	13.96 (3.10)	11	19.07 (2.61)	15	17.10 (3.47)	17

Seventeen parents (89 %) in the MBCT-P plus usual care arm provided consent to be interviewed and described feeling that the course met at least some of their expectations and that they had learnt skills that they could use in their daily lives. Parents described how these skills were able to help them with their own thoughts and emotions and with their parenting. The group experience was also described as acceptable. Parents reported feeling that they could be honest in the group sessions and that they could identify with the other members of the group. Parents also described some challenges, which included difficulty finding the time to do the daily homework practice and difficulty maintaining practice without the structure of the course.

#### Preliminary Efficacy of MBCT-P

Mean baseline BDI-II scores were in the mild range of symptoms, for both arms of the trial (13.0; 9.51). Parental stress scores were within the normal range (84.2; 77.81) (Abidin 1995). Ratings of children’s behaviour were slightly high when compared to normative data for 2–3 year olds and the mean for the usual care group was also high when considered against normative data for over 5 year olds (8.6; 10.3) (Meltzer et al. 2000; NHS GGC Information Services: unpublished, “SDQ for 2–4 year olds, normative data from Britain” 2014; Sim et al. 2013). Eleven participants (58 %) in the MBCT-P arm remained well compared to 6 (32 %) in the usual care arm (mean difference = 26 %; 95 % CI: −4 % to 57 %; p = 0.02).

The comparison of baseline scores for participants who did and did not complete the BDI-II, at the 4-month follow-up, demonstrated only a small difference in the mean for the usual care arm, with those who did not complete the follow-up scoring lower at baseline. This suggests that these participants not completing the 4-month follow-up could have resulted in a higher average score at 4 months for this arm. One participant in the MBCT-P arm did not complete the 4-month follow-up and had a higher baseline BDI-II score than those who completed the follow-up, meaning the 4-month average for the MBCT-P arm may have been lower than it would have been had all participants completed the follow-up. Table 4 shows the comparison of outcomes between MBCT-P and usual care.

**Table 4:** Comparison of outcomes between MBCT-P and usual care arms

Outcome	MBCT-P		Usual care		Crude difference	Adjusted mean difference			
	Mean (SD)	*n*	Mean (SD)	*n*	Estimate	Estimate	95 % CI	*p* value	Effect size
4-month follow-up									
Beck depression inventory	8.1 (9.1)	18	6.9 (9.0)	14	1.1	1.1	−5.6 to 7.9	0.7	0.1
Parenting stress index	73.2 (13.6)	13	84.1 (25.5)	9	−10.9	−9.6	−23.5 to 4.3	0.2	0.4
Strengths and difficulties	7.1 (3.0)	18	8.8 (4.0)	10	−1.7	−2.2	−4.2 to −0.3	0.03	0.6
Five facet mindfulness	127.6 (19.7)	18	124.3 (22.0)	11	3.3	10.2	−5.0 to 25.4	0.2	0.5
Self-compassion	17.35 (3.56)	18	13.96 (3.10)	11	3.4	3.3	0.6 to 6.0	0.2	1.1
9-month follow-up									
Beck depression inventory	4.6 (4.8)	17	11.3 (10.8)	16	−6.7	−7.0	−12.8 to −1.1	0.02	0.7
Parenting stress index	69.6 (13.4)	15	74.1 (15.9)	16	−4.5	−6.1	−16.2 to 4.1	0.2	0.4
Strengths and difficulties	7.7 (3.6)	17	10.3 (5.9)	16	−2.6	−1.4	−3.7 to 0.9	0.2	0.2
Five facet mindfulness	136.2 (17.6)	17	122.7 (25.1)	16	13.5	18.1	4.1 to 32.0	0.01	0.7
Self-compassion	19.07 (2.61)	15	17.10 (3.47)	17	1.97	2.9	1.0 to 4.8	0.005	0.8

Mean (SD) baseline BDI score for parents who were followed up at 4-month follow-up in MBCT-P arm = 12.22 (9.78); one parent not followed up in MBCT-P arm = 26.0; followed up in usual care arm = 10.71 (8.70); not followed up in usual care arm = 6.0 (5.61)

#### Therapist Adherence and Competence

Therapist adherence scores had a mean of 26.5 SD = 4.7, out of a total possible score of 34. This score is comparable to those originally reported for this scale and a subsequent trial of MBCT (Kuyken et al. 2008; Segal et al. 2002b). Therapist competency scores were in the ‘proficient’ and ‘advanced’ brackets across all six domains.

## Discussion

We adapted the existing MBCT course following feedback from parents. We used the early stages of the MRC Complex Interventions Framework (MRC 2008) to guide our treatment development work and prepare the ground for a definitive randomised controlled trial.

The adaptation of the length of the practices within the course was made due to parents feeling unable to complete longer practices. Difficulties with finding the time needed to practice has been highlighted in a previous qualitative study exploring parental participation in a mindfulness intervention (Allen et al. 2009) and therefore seemed an important change to make. Future research is needed to explore whether the completion of shorter practices impact upon the efficacy of the intervention.

The adaption of existing exercises into a parenting context enabled parents to incorporate them into their daily lives. Exercises, which were graded in difficulty, with the easiest being early on in the course, enabled parents to develop their skills over the course, in line with the existing MBCT course.

Participants reported that they found the MBCT-P content and group environment acceptable and attendance at sessions was good, suggesting good acceptability of the MBCT-P course. Barriers to participation included timing of the groups and changes in personal circumstances which were the reasons some parents randomised to the group could not attend. This improved when groups were scheduled for the evening. Retention was good suggesting that MBCT-P may also be a feasible intervention. The course may also result in a reduction in depressive symptoms and child psychopathology.

The majority of parents taking part in the trial attended half or more of sessions. Those who were interviewed also reported that the course met some, if not all, of their expectations, which suggests that MBCT-P was acceptable and feasible. Whilst it is not possible to draw firm conclusions about efficacy in a small pilot trial, these preliminary results suggest that participants randomised to MBCT-P have greater reduction in their depressive symptoms compared to usual care, over 9-month follow-up. There was also an increase in mindfulness and self-compassion levels, and an initial reduction in their child’s psychopathology at 4 months. Consistent with other parenting literature (cf. Scott et al. 2010), fathers were less likely to participate.

### Strengths and Limitations

Although the results described seem promising, there is some uncertainty about the true size of the effects due to the small sample size used. There was also a smaller number of parents who completed questionnaires in the usual care arm, meaning the results need to be interpreted cautiously. A larger trial will be needed to estimate the effects more precisely and to explore mechanisms of change.

We selected measures that have previously been shown to be sensitive to change and are widely used in the evaluation of parenting and mindfulness interventions. It is, however, important to consider the fact that all of the measures described were parental self-report and as such, there could be some shared method variance. Parents themselves were involved in the MBCT-P manual development to enhance the likelihood of it being acceptable and effective.

Despite these strengths, the sample was small and restricted in terms of educational level and the number of fathers who participated. It will be important to further test the accessibility of MBCT-P with people from a more diverse range of backgrounds and identify barriers to fathers’ participation. The study relied upon parental reports of children’s behaviour. Future research might usefully collect more objective data on parent-child interactions and children’s outcomes. Some of the researchers were also not blind to condition. In future work, it will be important to ensure that the whole research team are blind to allocation.

This work provides a first step towards the development of a course specifically aimed at preventing depressive relapse in parents of young children (Sawyer Cohen and Semple 2010). MBCT-P may be a helpful course for parents who experience recurrent depression, in terms of reducing depressive symptoms and parental stress and there is also some indication it may affect parental report of children’s behaviour; however, a larger trial would be needed to confirm if this is the case.

## References

[CR1] Abidin RR (1995). Parenting stress index.

[CR2] Allen M, Bromley A, Kuyken W, Sonnenberg SJ (2009). Participants’ experiences of mindfulness-based cognitive therapy: it changed me in just about every way possible. Behavioural and Cognitive Psychotherapy.

[CR3] Baer RA, Smith GT, Hopkins J, Krietemeyer J, Toney L (2006). Using self- report assessment methods to explore facets of mindfulness. Assessment.

[CR4] Baer RA, Smith GT, Lykins E, Button D, Krietemeyer J, Sauer S (2008). Construct validity of the five facet mindfulness questionnaire in meditating and nonmeditating samples. Assessment.

[CR5] Bailie C, Kuyken W, Sonnenberg S (2011). The experiences of parents in mindfulness-based cognitive therapy. Clinical Child Psychology and Psychiatry.

[CR6] Beck AT, Steer RA, Brown GK (1996). The Beck depression inventory—second edition.

[CR7] Bögels SM, Restifo K (2013). Mindful parenting: a guide for mental health practitioners.

[CR8] Bögels SM, Hoogstad B, Dun L, Schutter S, Restifo K (2008). Mindfulness training for adolescents with externalizing disorders and their parents. Behavioural and cognitive psychotherapy.

[CR9] Bögels SM, Hellemans J, Deursen S, Römer M, Meulen R (2014). Mindful parenting in mental health care: effects on parental and child psychopathology, parental stress, parenting, coparenting and marital functioning. Mindfulness.

[CR10] Bruin EI, Blom R, Smit FM, Steensal F, Bögel S (2015). MyMind: mindfulness training for youngsters with autism spectrum disorders and their parents. Autism.

[CR11] Coatsworth JD, Duncan LG, Greenberg MT, Nix RL (2010). Changing parent’s mindfulness, child management skills and relationship quality with their youth: results from a randomised pilot intervention trial. Journal of Child and Family Studies.

[CR12] Coatsworth JD, Duncan LG, Berrena E, Bamberger KT, Loeschinger D, Greenberg MT, Nix RL (2014). The mindfulness-enhanced strengthening families program: integrating brief mindfulness activities and parent training within an evidence-based prevention program. New Directions for Youth Development.

[CR13] Crane, R.S., Soulsby, J.G., Kuyken, W., Williams, J.M.G., Eames, C., Bartley, T., et al. (2012). The Bangor, Exeter & Oxford mindfulness-based interventions teaching assessment criteria (MBI-TAC). http://www.bangor.ac.uk/mindfulness/documents/MBI-TACMay2012.pdf Accessed 19^th^ February 2015.

[CR14] Crane RS, Eames C, Kuyken W, Hastings RP, Williams GJM, Bartley T, Evans A, Silverton S, Soulsby JG, Surawy C (2013). Development and validation of the mindfulness-based interventions teaching assessment criteria (MBI-TAC). Assessment.

[CR15] Davé S, Sheer L, Senior R, Nazareth I (2008). Associations between paternal depression and behaviour problems in children of 4–6 years. European Child and Adolescent Psychiatry.

[CR16] Evans, J.L. (2006). Parenting programmes: an important ECD intervention strategy. Paper Commissioned for the EFA Global Monitoring Report 2007, Strong foundations: early childhood care and education. http://unesdoc.unesco.org/images/0014/001474/147461e.pdf. Accessed 19^th^ February 2015.

[CR17] First, M.B., Spitzer, R.L., Gibbon, M., & Williams, J.B. (2002). *Structured clinical interview for DSM-IV-TR axis 1 disorders*, Research Version, Patient Edition with Psychotic Screen (SCID-I/P W/PSY SCREEN) Biometrics Research: New York State Psychiatric Institute.

[CR18] Goodman R (2001). The psychometric properties of the strengths and difficulties questionnaire (SDQ). Journal of the American Academy of Child and Adolescent Psychiatry.

[CR19] Goodman SH, Rouse MH, Connell AM, Broth MR, Hall CM, Heyward D (2011). Maternal depression and child psychopathology: a meta-analytic review. Clinical Child and Family Psychology Review.

[CR20] Hanington L, Ramchandani P, Stein A (2010). Parental depression and child temperament: assessing child to parent effects in a longitudinal population study. Infant Behaviour and Development.

[CR21] Hutchings J, Bywater T, Daley D, Gardner F, Whittaker C, Jones K (2007). Parenting intervention in Sure Start services for children at risk of developing conduct disorder: pragmatic randomised controlled trial. British Medical Journal.

[CR22] Kabat-Zinn K-Z (2008). Everyday blessings: the inner work of mindful parenting.

[CR23] Kuyken W, Byford S, Taylor RS, Watkins ER, Holden ER, White, Barrett B (2008). Mindfulness-based cognitive therapy to prevent relapse in recurrent depression. Journal of Consulting and Clinical Psychology.

[CR24] Lépine JP, Briley M (2011). The increasing burden of depression. Neuropsychiatric Disease and Treatment.

[CR25] Lovejoy MC, Graczyk PA, O’Hare E, Neuman G (2000). Maternal depression and parenting behaviour: a meta-analytic review. Clinical Psychology Review.

[CR26] Medical Research Council. (2008). *Developing and evaluating complex interventions: New guidance*: MRC.

[CR27] Meltzer H, Gatward R, Goodman R, Ford F (2000). Mental health of children and adolescents in Great Britain.

[CR28] National Research Council and Institute of Medicine (2009). Depression in parents, parenting and children: opportunities to improve identification, treatment and prevention.

[CR29] Neff KD (2003). The development and validation of a scale to measure self-compassion. Self and Identity.

[CR30] Office of National Statistics (2000). *Standard occupational classification 2000 volume 1: structure and descriptions of unit groups*. London: The Stationary Office. http://webarchive.nationalarchives.gov.uk/20160105160709/http://www.ons.gov.uk/ons/guide-method/classifications/archived-standard-classifications/standard-occupational-classification-2000/dissemination-media-and-availability/index.html. Accessed Oct 2009.

[CR31] Oord S, Bögels SM, Peijnenburg D (2012). The effectiveness of mindfulness training for children with ADHD and mindful parenting for their parents. Journal of Child and Family Studies.

[CR32] Piet J, Hougaard E (2011). The effect of mindfulness-based cognitive therapy for prevention of relapse in recurrent major depressive disorder: a systematic review and meta-analysis. Clinical Psychology Review.

[CR33] Ramchandani P, Stein A, Evans J, O’Connor TG, ALSPAC study team (2005). Paternal depression in the postnatal period and child development: a prospective population study. The Lancet.

[CR34] Ritchie J, Spencer L, O’Connor W, Ritchie J, Lewis J (2003). Carrying out qualitative analysis. Qualitative research practice: a guide for social research students and researchers (pp 219 – 262).

[CR35] Sawyer Cohen JA, Semple RJ (2010). Mindful parenting: a call for research. Journal of Child Family Studies.

[CR36] Scott S, Sylva K, Doolan M, Price J, Jacobs B, Crook C (2010). Randomised controlled trial of parent groups for child antisocial behaviour targeting multiple risk factors: the SPOKES project. Journal of Child Psychology and Psychiatry.

[CR37] Segal ZV, Williams JMG, Teasdale JD (2002). Mindfulness-based cognitive therapy for depression: a new approach to preventing relapse.

[CR38] Segal ZV, Teasdale JD, Williams JM, Gemar MC (2002). The mindfulness-based cognitive therapy adherence scale: inter-rater reliability, adherence to protocol and treatment distinctiveness. Clinical Psychology & Psychotherapy.

[CR39] Sharp D, Hay DF, Pawlby S, Schmucker G, Allen H, Kumar R (1995). The impact of postnatal depression on boys’ intellectual development. Journal of Child Psychology and Psychiatry.

[CR40] Sim F, O’Dowd J, Thompson L, Law J, Macmillan S, Affleck M, Gillberg C, Wilson P (2013). Language and social/emotional problems identified at a universal developmental assessment at 30 months. BMC Pediatrics.

[CR41] Velders FP, Dieleman G, Henrichs J, Jaddoe VW, Hofman A, Verhulst FC (2011). Prenatal and postnatal psychological symptoms of parents and family functioning: the impact on child emotional and behavioural problems. European Child and Adolescent Psychiatry.

[CR42] Weissman MM, Pilowsky DJ, Wickramaratne PJ, Talati A, Wisniewski SR, Fava M, STAR*D Child Team (2006). Remissions in maternal depression and child psychopathology: a STAR*D-child report. JAMA.

[CR43] Williams JM, Russell I, Russell D (2008). Mindfulness-based cognitive therapy: further issues in current evidence and future research. Journal of Consulting and Clinical Psychology.

[CR44] World Health Organisation Global Health Estimates. http://www.who.int/healthinfo/global_burden_disease/estimates/en/index2.html. Accessed 19^th^ February 2015.

